# An Intuitionistic Multiplicative ORESTE Method for Patients’ Prioritization of Hospitalization

**DOI:** 10.3390/ijerph15040777

**Published:** 2018-04-17

**Authors:** Cheng Zhang, Xingli Wu, Di Wu, Huchang Liao, Li Luo, Enrique Herrera-Viedma

**Affiliations:** 1 Business School, Sichuan University, Chengdu 610064, China; z_zhangcheng_c@163.com (C.Z.); xingliwusly@foxmail.com (X.W.); 2015141082036@stu.scu.edu.cn (D.W.); luolicc@163.com (L.L.); 2 Department of Computer Science and Artificial Intelligence, University of Granada, E-18071 Granada, Spain; viedma@decsai.ugr.es; 3 Faculty of Computing and Information Technology, King Abdulaziz University, Jeddah 21589, Saudi Arabia

**Keywords:** intuitionistic multiplicative preference relation, ORESTE, multiple criteria decision making, correlation coefficient, patients’ prioritization, hospital management

## Abstract

The tension brought about by sickbeds is a common and intractable issue in public hospitals in China due to the large population. Assigning the order of hospitalization of patients is difficult because of complex patient information such as disease type, emergency degree, and severity. It is critical to rank the patients taking full account of various factors. However, most of the evaluation criteria for hospitalization are qualitative, and the classical ranking method cannot derive the detailed relations between patients based on these criteria. Motivated by this, a comprehensive multiple criteria decision making method named the intuitionistic multiplicative ORESTE (organísation, rangement et Synthèse dedonnées relarionnelles, in French) was proposed to handle the problem. The subjective and objective weights of criteria were considered in the proposed method. To do so, first, considering the vagueness of human perceptions towards the alternatives, an intuitionistic multiplicative preference relation model is applied to represent the experts’ preferences over the pairwise alternatives with respect to the predetermined criteria. Then, a correlation coefficient-based weight determining method is developed to derive the objective weights of criteria. This method can overcome the biased results caused by highly-related criteria. Afterwards, we improved the general ranking method, ORESTE, by introducing a new score function which considers both the subjective and objective weights of criteria. An intuitionistic multiplicative ORESTE method was then developed and further highlighted by a case study concerning the patients’ prioritization.

## 1. Introduction

**Figure 1:**
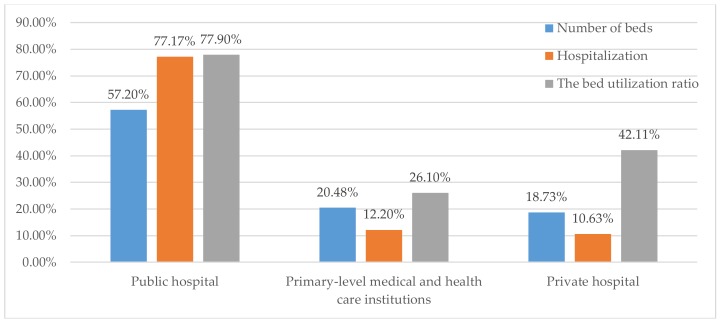
Distribution of medical resources in different types of hospital in Chengdu, China.

Nowadays, with the rapid growth of the world population, finite medical resources make it hard to meet people’s requirements for healthcare. Most countries face the issue of how to rationally and effectively allocate medical resources so that patients can be treated timely. The same situation exists in China. The scales of public hospitals in China are far beyond that of the primary-level medical and health care institutions. Figure 1 illustrates the significant difference between different types of hospitals in Chengdu, China. It shows that the number of beds in public hospitals are about 1.33 times those in other types of hospitals. As we know, there are many high quality medical resources in public hospitals, such as advanced medical equipment and high-quality top-notch medical personnel. Patients would prefer to go to the public hospitals rather than the primary-level medical or healthcare institutions. The large-scale general hospitals are overcrowded, while the primary medical institutions are not. How to determine the patients’ order of hospitalization for a public hospital with tight beds has become a key issue.

Patients’ prioritization of hospitalization can be taken as a Multiple Criteria Decision Making (MCDM) problem, which consists of (1) evaluating the patients’ performances; (2) determining the weights of evaluation criteria; (3) aggregating the evaluation values with respect to the criteria weights and then determining the patients’ orders. Due to the complexity of objective things, usually it is hard to evaluate the utility value of an alternative (criterion) directly. However, giving the preference relation of pairwise alternatives (criteria) is easy to implement. Fuzzy set theory [1] can be taken as a useful tool to represent the complex preference information in decision making problems [2], and it has been extended to various forms, such as extended hesitant fuzzy set [3], linguistic intuitionistic fuzzy [4], hesitant linguistic intuitionistic fuzzy set [5], and intuitionistic fuzzy set [6]. Orlovsky [7] developed the fuzzy preference relation to describe the dominance degree of alternative $A_{i}$ over $A_{k}$ under criterion $c_{j}$ by a membership degree $\rho_{ik}^{j}$. Given the ambiguity and uncertainty of people’s cognition, Xu [8] depicted the preference degree of pairwise alternatives by both membership function and non-membership function and expressed it as $\left(\rho_{ik}^{j-},1-\rho_{ik}^{j+}\right)$. Besides, there are some other preference relations which have been researched in recent years, such as the single-valued trapezoidal neutrosophic preference relation [9] and the 2-tuple fuzzy linguistic preference relation [10]. However, these models are limited in the 0.1−0.9 scale. It deems the relation between preference degree of $A_{i}$ over $A_{k}$ and the preference degree of $A_{k}$ over $A_{i}$ being complementary. That is to say, $\rho_{ik}^{j-}+\rho_{ki}^{j-}=1$ if there is no hesitation. In fact, the preference relation of pairwise objects is the reciprocal relationship, which implies $\rho_{ik}^{j-}\times \rho_{ki}^{j-}=1$ if there is no hesitation. Therefore, Xia et al. [11] employed the Saaty’s 1/9−9 scale [12] instead of the 0.1−0.9 scale to describe the pairwise preference relation, and proposed the concept of the Intuitionistic Multiplicative Preference Relation (IMPR). 

The IMPR model is flexible in expressing the uncertain preference opinions of individuals given that it can reflect the membership, non-membership, and hesitation degree clearly. Because of the effectiveness, IMPR has attracted growing concerns. Researchers studied the operations and comparison method [13], measurements [14,15], consensus models [16], and aggregation models [17,18] of intuitionistic multiplicative sets (IMSs). Furthermore, the IMPR has been combined with different weighting methods and ranking techniques to handle the MCDM problems. Ren et al. [19] extended the classical analytic hierarchy process to solve the MCDM with the IMPRs, and applied it to deal with the evaluation of site section for hydropower station. To overcome the questionable consistency checking and repairing method in Ren et al. [19], Zhang et al. [20] developed a new intuitionistic multiplicative group analytic hierarchy process based on the consistency concept proposed in Ref. [21]. Mou et al. [22] extended the best-worst method to the uncertain environment with the evaluation expressed as IMPR and applied it to solve the problem concerning medical diagnosis of patients with emphysema.

However, there are some drawbacks in the exiting MCDM methods with IMPRs. (1) The connection between criteria is ignored in the existing IM-MCDM methods and the highly-related criteria may mislead the results; (2) The uncertain preference opinions of experts on the importance of criteria are translated into fuzzy criteria weights by some weighting techniques, such as the possibility degree-based weighting method [19,20] and the optimization model-based weighting method [22]. Information loss may be caused by these translating processes; (3) They cannot distinguish the indifference and incomparability relations between two alternatives. In this regard, the results lack reliability.

The ORESTE [23] is a general ranking method to deal with the MCDM problems with qualitative information. It first determines the weak ranking of alternatives by aggregating criterion weights and alternatives’ performance which are expressed as orders. Then, the preference, indifference, and incomparability (PIR) relations between pairwise alternatives are distinguished by conflict analysis. Finally, a strong ranking can be obtained to clearly reveal the relationships between the alternatives. Compared with other decision-making methods, such as PROMETHEE, TOPSIS, and VIKOR methods, with ORESTE it is not necessary to translate the fuzzy importance degrees evaluated by experts to crisp weights. In this sense, it can avoid information loss produced in the translating process. Both the TOPSIS and VIKOR methods cannot derive the indifference relation between alternatives in application. A strict rule exists in the conflict analysis of the PROMETHEE method, such as the two alternatives are indifference relation if and only if both their positive and negative outranking flows are equal. In this sense, the derived rankings of alternatives by the PROMETHEE method may be unconvincing. However, all the above mentioned problems can be carried out by the ORESTE method. The ORESTE method has been applied in many fields, such as web design firm selection [24], insurance company selection [25], ports’ ranking [26] and innovative design selection of shared cars [27]. Thus, in this paper, we apply the ORESTE method to explore the problem of patients’ prioritization of hospitalization.

To solve the problem of patients’ prioritization for hospitalization, staff in hospital need to consider multiple complex evaluation criteria, and then give their ranking results of patients through a complex analysis process. As usual, traditional methods are not only difficult for evaluating the criteria, such as the value of pathology, clinical features, related risks, and the emergency degree, but also hard for deriving scientific ranking results of patients. Thus, this paper aims to propose a comprehensive method to handle the MCDM problems with intuitionistic multiplicative information, and then apply it to solve the problem concerning patients’ prioritization of hospitalization in HX hospital.

There are some limitations with the traditional ORESTE method, i.e., it is limited in considering the subjective and objective weights of criteria simultaneously [25,26]. Besides, it is limited in handling the evaluations expressed as IMPRs. Therefore, we concentrate on overcoming the defects mentioned above as well as deriving robust decision results. The paper is highlighted by the following contributions:

1. We present a correlation coefficient-based weight determining method in the context of IMPR. This method can avoid highly-related criteria misleading the final results by assigning small weights to them.
2. We introduce a new global score function to aggregate the weights of criteria and the alternatives’ performance under each criterion. Both the subjective and objective weights of criteria are considered in this function.
3. We derive threshold values which can be used to determine the PIR relations between alternatives under the IMPR environment.
4. We carry out a case study concerning patients’ prioritization for hospitalization in a public hospital in China by the proposed IM-ORETSE method.

The rest of this paper is summarized as follows: Section 2 reviews the knowledge of IMPR and the traditional ORESTE method. The IM-ORESTE method is proposed in Section 3. In Section 4, we present a case study concerning the patients’ prioritization for hospitalization to illustrate the application of our proposed method. The paper ends with some interesting conclusions in Section 5.

## 2. Preliminaries

In this section, some basic knowledge of IMPR and the ORESTE method are introduced.

### 2.1. Intuitionistic Multiplicative Preference Relation

Xia et al. [11] proposed the definition of IMPR.

**Definition** **1.** [11] *Let X be fixed. An intuitionistic multiplicative set (IMS) in X is defined as*:

(1) D={<x, (ρ(x), σ(x))> | x∈X}

*which assigns to each element x a membership function ρ(x) and a non-membership function σ(x), with the conditions: 1/9≤ρ(x), σ(x)≤9, ρ(x)σ(x)≤1, ∀x∈X.*

For convenience, the pair (ρ(x), σ(x)) is called an Intuitionistic Multiplicative Number (IMN). For each IMN, τ(x)=1/ρ(x)σ(x) can be described as uncertain or hesitant information. Obviously, $1/9^{2}\leq \tau (x)\leq 9^{2}$, ∀x∈X. An IMN can also be denoted as (ρ(x), σ(x),τ(x)).

Let $A=\{A_{1},A_{2},\cdots ,A_{m}\}$ be m objects. An IMPR is expressed as $X={\left(\alpha_{ik}\right)}_{m\times m}$ where $\alpha_{ik}=(\rho_{ik},\sigma_{ik})$ is an IMN. $\rho_{ik}$ expresses the degree to which object $A_{i}$ is preferred to $A_{k}$, and $\sigma_{ik}$ expresses the degree to which object $A_{i}$ is not preferred to $A_{k}$. They meet the conditions $\rho_{ik}=\sigma_{ki}$, $\sigma_{ik}=\rho_{ki}$, $\rho_{ik}\sigma_{ik}\leq 1$ and $1/9\leq \rho_{ik},\;\sigma_{ik}\leq 1$.

**Definition** **2.** [11] *For an IMN $\alpha =(\rho_{\alpha },\;\sigma_{\alpha })$, the score function of α is defined as*

(2) $$s(\alpha )={\rho_{\alpha }/\sigma }_{\alpha }$$

*and the accuracy function is $h(\alpha )=\rho_{\alpha }\sigma_{\alpha }$. For two IMNs $\alpha_{1}$ and $\alpha_{2}$*:

- If $s(\alpha_{1})>s(\alpha_{2})$, then $\alpha_{1}>\alpha_{2}$.
- If $s(\alpha_{1})=s(\alpha_{2})$, then (i) If $h(\alpha_{1})>h(\alpha_{2})$, then $\alpha_{1}>\alpha_{2}$; (ii) If $h(\alpha_{1})=h(\alpha_{2})$, then $\alpha_{1}=\alpha_{2}$.

Qian and Niu [28] defined some operations of IMNs, which can guarantee the closeness of operations.

**Definition** **3.** [28] *Let $\alpha_{1}=(\rho_{1},\;\sigma_{1})$ and $\alpha_{2}=(\rho_{2},\;\sigma_{2})$ be two IMNs, then*

*(1)*  $\alpha_{1}⊕\alpha_{2}=\left(9^{\frac{\log_{9}\left(\rho_{\alpha 1}\rho_{\alpha 2}\right)-\log_{9}\rho_{\alpha 1}\log_{9}\rho_{\alpha 2}+1}{2}},9^{\frac{\log_{9}\left(\sigma_{\alpha 1}\sigma_{\alpha 2}\right)+\log_{9}\sigma_{\alpha 1}\log_{9}\sigma_{\alpha 2}-1}{2}}\right)$;
*(2)*  $\lambda \alpha =\left(9^{1-2{\left(\frac{1-\log_{9}\rho_{\alpha }}{2}\right)}^{\lambda }},9^{2{\left(\frac{1+\log_{9}\sigma_{\alpha }}{2}\right)}^{\lambda }-1}\right)$, where λ>0 is a real number.

Additionally, to measure the deviation between IMSs, Jiang et al. [14] proposed the distance measure between IMSs as

(3) $$d(A,B)=\frac{1}{4}\sum_{i=1}^{n}\left(\left|\log_{9}\frac{\rho_{A}\left(x_{i}\right)}{\rho_{B}\left(x_{i}\right)}\right|+\left|\log_{9}\frac{\sigma_{A}\left(x_{i}\right)}{\sigma_{B}\left(x_{i}\right)}\right|+\left|\log_{9}\frac{\tau_{A}\left(x_{i}\right)}{\tau_{B}\left(x_{i}\right)}\right|\right)$$

### 2.2. The ORESTE Method

The ORESTE was first proposed by Roubens [23] and further improved by Pastijn and Leysen [29]. In ORESTE, both the importance degrees of criteria and the evaluations of alternatives are expressed with rankings. Compared with other ranking methods, the decision results of ORESTE are not the single ranking order but the PIR relations among alternatives. In ORESTE, experts are invited to evaluate the initial ranking, $r_{j}$ of criterion $c_{j}$ based on its importance degree and the initial ranking $r_{ij}$ of $A_{i}$ under $c_{j}$. The steps of the ORESTE method are shown as follows:

**Algorithm** **1** (The ORESTE method)

**Step 1.** Calculate the global preference score of alternative $A_{i}$ under criterion $c_{j}$ by Equation (4). (See Refs. [23,29] for more details)

(4) $$D_{ij}=\sqrt{\varsigma r_{j}{}^{2}+\left(1-\varsigma \right)r_{ij}{}^{2}}$$

where ς indicates the relative importance degree between the ranks of alternatives and criteria in final decision. The global weak ranking $r(A_{ij})$ of each alternative is derived by $D_{ij}$ in ascending order. If $D_{ij}>D_{kj}$, then $r(A_{ij})>r(A_{kj})$; if $D_{ij}=D_{kj}$, then $r(A_{ij})=r(A_{kj})$.

**Step 2.** Aggregate the global weak ranking of each alternative by Equation (5)

(5) $$R\left(A_{i}\right)=\sum_{j=1}^{n}r(A_{ij})$$

The weak rankings of alternatives are determined by the ascending order of $R\left(A_{i}\right)$.

**Step 3.** Determine the PIR relations between alternatives after the conflict analysis. If the weak rankings of two alternatives are equal or similar, they are not always the indifference relation that they can replace to each other in decision making. Two alternatives may have the same weak rankings but their performances are quite different under some criteria. In this case, if we select one of them, the criteria under which the selected alternative performs well than another will be highlighted. Therefore, it is necessary to distinguish the incomparability relation from the indifference relation between two alternatives. The ORESTE method applies the preference intensities to make the conflict analysis.

The average preference intensity of $A_{i}$ over $A_{k}$ is:

(6) $$T\left(A_{i},A_{k}\right)=\sum_{j=1}^{n}\left(\max\left(r(A_{kj})-r(A_{ij}),0\right)/\left(m-1\right)n^{2}\right)$$

The net preference intensity of $A_{i}$ over $A_{k}$ is:

(7) $$\Delta T\left(A_{i},A_{k}\right)=T\left(A_{i},A_{k}\right)-T\left(A_{k},A_{i}\right)$$

The conflict analyses to construct the PIR structures are as follows:

1. If |ΔT|≤β and $\min\left(T\left(A_{i},A_{k}\right),T\left(A_{k},A_{i}\right)<T^{*}\right)$, then $A_{i}\;I\;A_{k}$;
2. If $\min\left(T\left(A_{i},A_{k}\right),T\left(A_{k},A_{i}\right)\right)/\left|\Delta T\right|\geq \gamma$, then $A_{i}\;R\;A_{k}$;
3. If |ΔT|>β, $\min\left(T\left(A_{i},A_{k}\right),T\left(A_{k},A_{i}\right)\right)/\left|\Delta T\right|<\gamma$ and $\Delta T\left(A_{i},A_{k}\right)>0$, then $A_{i}\;P\;A_{k}$;
4. If |ΔT|>β, $\min\left(T\left(A_{i},A_{k}\right),T\left(A_{k},A_{i}\right)\right)/\left|\Delta T\right|<\gamma$ and $\Delta T\left(A_{i},A_{k}\right)<0$, then $A_{k}\;P\;A_{i}$. where β, γ, and $T^{*}$ are three thresholds to distinguish the PIR relations. Their values are determined by
  (8) $$\beta <1/\left(m-1\right)n,\;\gamma >\left(n-2\right)/4,\;T^{*}<\chi /2\left(m-1\right)$$
  where χ is given by DMs subjectively to distinguish the indifference or incomparability relation between two alternatives under each criterion. It denotes the maximal rank difference between two indifferent alternatives. In other words, $A_{i}$ is indifferent to $A_{k}$ if the ranking difference of them is small than χ with respect to criterion $c_{j}$.

**Step 4.** Determine the strong ranking by the weak ranking and the PIR structure.

As a general outranking method, the ORESTE has some advantages: (1) It is simple to understand and easy to make application in terms of technical parameters which are deduced objectively [24]. (2) The process is clear and visible in that we can observe the changes in result when the initial evaluations change or different thresholds are predefined. (3) It does not have to translate the fuzzy criteria weights to crisp weights, which can avoid information loss. Huylenbroeck [30] illustrated that the conflict analysis is more effective in separating the PIR relations than the PROMETHEE and ELECTRE. Based on the flexible manufacturing system selection problem, Chatterjee and Chakraborty [31] validated the superiority of the ORESTE method over five acceptable outranking methods in terms of the reliability of results.

## 3. Intuitionistic Multiplicative ORESTE Method

This section develops an IM-ORESTE method to solve the MCDM problems with the evaluations expressed as IMNs. We first present an objective weight determining method based on the correlation coefficient, which can avoid the misleading results caused by highly-related criteria. Then, the classical ORESTE method is improved by introducing a new global score function. Finally, the IM-ORESTE method is proposed based on the distance measure between IMNs.

### 3.1. Description of the Intuitionistic Multiplicative MCDM Problem

A MCDM problem consists of the alternatives $\left\{A_{1},\cdots ,A_{i},\cdots ,A_{m}\right\}$, the criteria $\left\{c_{1},\cdots ,c_{j},\cdots ,c_{n}\right\}$ and their weights $\left(\omega_{1},\cdots ,\omega_{j},\cdots ,\omega_{n}\right)$. The performances of pairwise alternatives are compared by experts under each criterion. The preference of $A_{i}$ over $A_{k}$ under $c_{j}$ is expressed as an IMN, $\alpha_{ik}^{j}$. We can establish the decision matrices as follows:

(9) $$D^{j}=\begin{array}{c} A_{1}\\ \vdots \\ A_{i}\\ \vdots \\ A_{m} \end{array}\;\;\;\;\left[\begin{array}{ccccc} \alpha_{11}^{j} & \cdots & \alpha_{1i}^{j} & \cdots & \alpha_{1m}^{j}\\ \vdots & \ddots & \vdots & \ddots & \vdots \\ \alpha_{i1}^{j} & \cdots & \alpha_{ii}^{j} & \cdots & \alpha_{im}^{j}\\ \vdots & \ddots & \vdots & \ddots & \vdots \\ \alpha_{m1}^{j} & \cdots & \alpha_{mi}^{j} & \cdots & \alpha_{mm}^{j} \end{array}\right],\;j=1,2,\cdots ,n$$

There are two types of criteria weights to describe the importance of criteria, including the subjective weights and the objective weights. The subjective weights are determined by experts’ preferences. The relative importance evaluated by experts of pairwise criteria are expressed as IMNs, $\alpha^{jt}$, j,t=1,2,⋯,n. The objective weights are determined by the distribution of evaluation values under each criterion. Considering both types of weights can derive a robust ranking of alternatives.

### 3.2. The Correlation Coefficient-Based Weighting Method

This part presents an objective weight determining method based on the correlation coefficient of criteria with the IMNs information.

Most practical decision-making problems are defined on multiple criteria. The “conflict” is the fundamental concept in MCDM problems in that different criteria should provide independent information on the performance of alternatives [32]. However, there are always highly interactive influence, strong repeatability, and linear crossover among criteria because of the interrelated and interactive objective things. For example, when evaluating a teacher’s ability, the criterion, respecting the dignity of students, has a causative effect on the criterion, students’ satisfaction. If the same weights are assigned to these highly-related criteria, the results will be biased, because the same information existing in more than one criterion is reconsidered when aggregating the performance of each alternative under all criteria. Merging interdependent criteria by some methods, such as principle component analysis [33], is complex and may mislead the final results. However, deleting these criteria is impolite and may lose useful information to some extent. Therefore, assigning a small weight to the highly-related criterion is a reasonable way to overcome this problem [32]. Inspired by this idea, we used the correlation coefficient to denote the correlation degree of one criterion to others. The larger the correlation coefficient is, the smaller is the weight of the criteria. The steps of the correlation coefficient-based weighting method are constructed as follows:

**Step 1.** Aggregate the preference values $\alpha_{ik}^{j}$ (k=1,2,⋯,m) of alternative $A_{i}$ compared with others $A_{k}$ (k=1,2,⋯,m； k≠i) under criterion $c_{j}$.

(10) $$\alpha_{i}^{j}=\frac{1}{m-1}\overset{m}{\underset{k=1}{⊕}}\alpha_{ik}^{j},\;k\neq i$$

where $\alpha_{i}^{j}$ is an IMN of alternative $A_{i}$ with respect to criterion $c_{j}$. The IM decision matrix can be established as $D={\left(\alpha_{i}^{j}\right)}_{m\times n}$.

**Step 2.** Calculate the Pearson correlation coefficient between criteria $c_{j}$ and $c_{t}$ based on the distance measure of IMNs.

(11) $$C_{jt}=\frac{\sum_{i=1}^{m}\left[\left(\frac{d_{ij}}{\underset{i}{\max}(d_{ij})}-\frac{1}{m}\sum_{i=1}^{m}\frac{d_{ij}}{\underset{i}{\max}(d_{ij})}\right)\times \left(\frac{d_{it}}{\underset{i}{\max}(d_{it})}-\frac{1}{m}\sum_{i=1}^{m}\frac{d_{it}}{\underset{i}{\max}(d_{it})}\right)\right]}{\sqrt{\sum_{i=1}^{m}{\left(\frac{d_{ij}}{\underset{i}{\max}(d_{ij})}-\frac{1}{m}\sum_{i=1}^{m}\frac{d_{ij}}{\underset{i}{\max}(d_{ij})}\right)}^{2}}\times \sqrt{\sum_{i=1}^{m}{\left(\frac{d_{it}}{\underset{i}{\max}(d_{it})}-\frac{1}{m}\sum_{i=1}^{m}\frac{d_{it}}{\underset{i}{\max}(d_{it})}\right)}^{2}}}$$

where $d_{ij}=d(\alpha_{i}^{j},\alpha_{}^{j+})$, $d_{it}=d(\alpha_{i}^{t},\alpha_{}^{t+})$.

**Step 3.** Compute the subjective weights by:

(12) $$\omega_{j}^{\prime }=\sum_{t=1}^{n}\left(1-C_{jt}\right)/\sum_{j=1}^{n}\left(\sum_{t=1}^{n}\left(1-C_{jt}\right)\right)$$

There are other kinds of objective weighting methods, such as the standard deviation-based weighting method [34] and the entropy measure-based weighting method [35]. They are based on the dispersion degrees of evaluations in that the criterion with great dispersion degree of alternatives’ performances is assigned a big weight. They have less influence on the final rankings, since the gaps between alternatives are widened. More importantly, however, they fail to handle the highly-related criteria. In the following, we take a simple example to illustrate the effectiveness of our proposed method.

**Example** **1.** *Suppose that we rank three teachers $A_{1}$, $A_{2}$, and $A_{3}$ based on respecting the dignity of students $c_{1}$, students’ satisfaction $c_{2}$ and stimulating students’ initiative $c_{3}$. The preference matrices are*

$$D^{1}=\left[\begin{array}{ccc} \left(1,1\right) & \left(1/4,4\right) & \left(1/3,3\right)\\ \left(4,1/4\right) & \left(1,1\right) & \left(3/2,2/3\right)\\ \left(3,1/3\right) & \left(2/3,3/2\right) & \left(1,1\right) \end{array}\right],\;D^{2}=\left[\begin{array}{ccc} \left(1,1\right) & \left(1/4,4\right) & \left(1/3,3\right)\\ \left(4,1/4\right) & \left(1,1\right) & \left(3/2,2/3\right)\\ \left(3,1/3\right) & \left(2/3,3/2\right) & \left(1,1\right) \end{array}\right],\;D^{3}=\left[\begin{array}{ccc} \left(1,1\right) & \left(4,1/4\right) & \left(3/2,2/3\right)\\ \left(1/4,4\right) & \left(1,1\right) & \left(1/3,3\right)\\ \left(2/3,3/2\right) & \left(3,1/3\right) & \left(1,1\right) \end{array}\right]$$

We can find that criteria $c_{1}$ and $c_{2}$ are highly correlated since similar information is presented by them. By Equation (10), we obtain the IM decision matrix as:

$$D=\left[\begin{array}{ccc} \left(961/3319,3319/961\right) & \left(961/3319,3319/961\right) & \left(1189/441,441/1189\right)\\ \left(1189/441,441/1189\right) & \left(1189/441,441/1189\right) & \left(961/3319,3319/961\right)\\ \left(4083/2461,2461/4083\right) & \left(4083/2461,2461/4083\right) & \left(4083/2461,2461/4083\right) \end{array}\right]$$

The scores $s(\alpha_{i}^{j})$ (i=1,2,⋯m, j=1,2,⋯n) calculated by Equation (2) are shown as

$$s=\left[\begin{array}{ccc} 0.0838 & 0.0838 & 7.2692\\ 7.2692 & 7.2692 & 0.0838\\ 2.7526 & 2.7526 & 2.7526 \end{array}\right]$$

We rank the alternatives based on the scores of IMNs. The comprehensive score of each alternative can be calculated by:

(13) $$s(A_{i})=\prod_{j=1}^{n}s{\left(\alpha_{i}^{j}\right)}^{\omega_{j}}$$

In the following, we use two methods to calculate the objective weights of criteria and then derive the rankings of the three teachers.

1. Rank the teachers based on the correlation coefficient-based weighting method
  From Equations (3)–(5), we obtain the weights of criteria are $\omega_{1}^{\prime }=0.25$, $\omega_{2}^{\prime }=0.25$, and $\omega_{3}^{\prime }=0.5$. By Definition 2, we obtain $s(A_{1})=0.97$, $s(A_{2})=0.97$, and $s(A_{3})=2.02$, thus $A_{3}≻A_{2}=A_{1}$.
2. Rank the teachers based on the standard deviation-based weighting method
  The dispersion degree-based weighting methods aim to determine the criteria weights based on the variation of evaluations under each criterion. A small weight is assigned when the evaluations under this criterion are close, while a big weight is assigned when the gaps of the evaluations are large. We use a representative method of this type, the standard deviation-based weighting method, to solve Example 1. Standard deviation $\sigma_{j}$ under IM context can be defined as:
  (14) $$\sigma_{j}=\sqrt{\frac{\sum_{i=1}^{m}{\left((s(\alpha_{i}^{j})-1/m\sum_{i}^{m}s(\alpha_{i}^{j})\right)}^{2}}{m}}$$

In this Example, by Equation (14), we obtain $\sigma_{1}=1.65$, $\sigma_{2}=1.65$, and $\sigma_{3}=1.65$. After normalization, we obtain ${\tilde{\omega }}_{1}=0.33$, ${\tilde{\omega }}_{2}=0.33$, and ${\tilde{\omega }}_{3}=0.33$. From Definition 2, we get $\tilde{s}(A_{1})=0.59$, $s(A_{2})=1.58$, and $s(A_{3})=2$, thus $A_{3}>A_{2}>A_{1}$. The same weight is assigned to each criterion since these criteria maintain the same variation of evaluations. 

Comparative analyses: From the preference matrices, we can find that $c_{1}$ is highly related to $c_{2}$ since similar information is composed in these two criteria. We can integrate them as one criterion to describe the performance of alternatives. By the correlation coefficient-based weighting method, small weights are assigned to them. The correlation coefficient-based weighting method is effective in avoiding the misleading results caused by the highly-related criteria. We find that $A_{2}≻A_{1}$ are derived by the standard deviation-based weighting method. However, both alternatives perform equally in total since one of $c_{1}$ and $c_{2}$ should be considered in decision making in this case. We can conclude that the misleading results caused by some highly-related criteria cannot be avoided by the dispersion degree-based weighting methods.

### 3.3. The IM-ORESTE Method

This part improves the ORESTE method by introducing the objective criteria weights and extends it to solve the MCDM problems with the evaluation values expressed as IMNs. In IM-ORESTE, both the preferences on criteria evaluated by experts subjectively and the preferences on pairwise alternatives under each criterion are expressed as IMNs. Like the classical ORESTE method, the process of the IM-ORESTE method is divided into two stages.

**Stage 1.** Determine the weak rankings

We introduce a global preference score function to consider both the subjective and objective weights of criteria. It is hard to assign a crisp weight to a criterion by experts due to the fuzziness of people’s cognition and the complexity of the objects. However, it is easy to compare the importance between two criteria. The IMN is effective in describing the experts’ uncertain and fuzzy preferences over criteria. Suppose that the preference of $c_{j}$ over $c_{t}$ is denoted as $\alpha^{jt}$ (j,t=1,2,⋯n). From Equation (10), we obtain the IMN $\alpha^{j}$ of each criterion and denote it as the fuzzy subjective weight $\omega_{j}^{\prime \prime }$. Translating the fuzzy subjective weights to the crisp numbers will cause information loss. Thus, we are not supposed to integrate the subjective and objective weights of criterion into a collective one. Motivated by the global score function proposed in Ref. [29], we aggregate each alternative’s performance and subjective weights of criteria by the weighted Euclidean distance measure of IMNs. Furthermore, considering the objective weights, we introduce a new global score function as:

(15) $$GS_{ij}=\omega_{j}^{\prime }\sqrt{\xi {\left(d\left(\alpha_{i}^{j},\alpha_{i}^{j+}\right)\right)}^{2}+(1-\xi ){\left(d\left(\omega_{j}^{\prime \prime },\omega_{j+}^{\prime \prime }\right)\right)}^{2}}$$

where $\omega_{j+}^{\prime \prime }=\underset{j}{\max}\omega_{j}^{\prime \prime }=\underset{j}{\max}\left\{\alpha^{j}\right\}$, ξ indicates the relative importance between the alternative’s performance and the criterion importance in calculating the global preference score of $A_{i}$ under $c_{j}$. ξ∈(0,1] and $GS_{ij}\in [0,1]$.

The utility value of each alternative is determined by aggregating the global scores under all criteria:

(16) $$U_{i}=\sum_{j=1}^{n}GS_{ij}$$

The weak rankings are determined by the utility values in ascending order.

**Stage 2.** Determine the PIR relation

It is a strict way to determine the relations among alternatives based on the utility values. If $U_{i}<U_{k}$, then $A_{i}>A_{k}$; if $U_{i}=U_{k}$, then $A_{i}=A_{k}$. However, there is usually a certain amount of error in our evaluations due to the fuzziness of thinking and the limitation of cognition. Therefore, we are supposed to allow a certain range of differences when comparing the two alternatives’ utility values. The utility value is limited to derive the definitive relationship between two alternatives. To overcome this defect, we further conduct the pairwise comparison under each criterion based on the global scores. Compared with the initial preference relation between two alternatives given by experts directly, the global scores integrate criteria weights information. The preference intensity of $A_{i}$ over $A_{k}$ with respect to $c_{j}$ can be defined as:

(17) $$PI_{j}(A_{i},A_{k})=max\left\{\left(GS_{kj}-GS_{ij}\right),0\right\}$$

$PI_{j}(A_{i},A_{k})$ indicates the superiority of $A_{i}$ over $A_{k}$ with respect to $c_{j}$, and $PI_{j}(A_{i},A_{k})\in [0,1]$. The comprehensive preference intensity of $A_{i}$ over $A_{k}$ under all criteria can be calculated as:

(18) $$PI(A_{i},A_{k})=\sum_{j=1}^{n}PI_{j}(A_{i},A_{k})$$

$PI(A_{i},A_{k})$ indicates the comprehensive superiority of $A_{i}$ over $A_{k}$, and $PI(A_{i},A_{k})\in [0,1]$. The net preference intensity of $A_{i}$ over $A_{k}$ can be defined as

(19) $$\Delta PI(A_{i},A_{k})=PI(A_{i},A_{k})-PI(A_{k},A_{i})$$

$\Delta PI(A_{i},A_{k})$ determines the overall preference relation between $A_{i}$ and $A_{k}$. $\Delta PI(A_{i},A_{k})\in [-1,1]$, and it satisfies $\Delta PI(A_{i},A_{k})=U_{k}-U_{i}$ and $\left|\Delta PI(A_{i},A_{k})\right|=$ $\left|\Delta PI(A_{k},A_{i})\right|$.

The PIR relations between two alternatives should meet the following conditions:

(1) When the absolute value of the net preference intensity $\left|\Delta PI(A_{i},A_{k})\right|$ is large enough, we can ensure that $A_{i}$ and $A_{k}$ are preference relation. That is, $A_{i}\;P\;A_{k}$ or $A_{k}\;P\;A_{i}$ if $\left|\Delta PI(A_{i},A_{k})\right|\geq \mu$.

(2) If $\left|\Delta PI(A_{i},A_{k})\right|<\mu$, and the performances of $A_{i}$ and $A_{k}$ are similar under each criterion, that is to say, $\max(PI_{j}(A_{i},A_{k}),PI_{j}(A_{k},A_{i}))<\delta$, then $A_{i}$ and $A_{k}$ is the indifference relation $A_{i}\;I\;A_{k}$. In this situation, they can replace each other when making a decision.

(3) If $\left|\Delta PI(A_{i},A_{k})\right|<\mu$, but the performances of $A_{i}$ and $A_{k}$ are quite different under some criteria, that is to say, $\max(PI_{j}(A_{i},A_{k}),PI_{j}(A_{k},A_{i}))\geq \delta$, then $A_{i}$ and $A_{k}$ is the incomparability relation $A_{i}\;R\;A_{k}$. In this case, they cannot replace each other when making a decision. For example, we suppose that two products, $A_{1}$ and $A_{2}$, are opposite with regard to quality and price. If $A_{1}$ is better than $A_{2}$ in quality, then $A_{2}$ is better than $A_{1}$ in price. If quality and price have the same weight, $A_{1}$ and $A_{2}$ are not the preference relation but the indifference relation. If we select $A_{1}$, the quality is highlighted, while if we select $A_{2}$, the price is highlighted. In this condition, we need to redefine the importance of the criteria to select $A_{1}$ or $A_{2}$.

We define μ as the preference threshold and δ as the indifference threshold. We need to determine reasonable values of μ and δ, respectively. It is not easy to assign the value of δ based on the preference intensity $PI_{j}(A_{i},A_{k})$. Therefore, we employ the initial preference values $\alpha_{ik}^{j}$ given by experts to deduce the value of δ. In this way, δ is objective and meets our cognition. 

Suppose that there are one criterion and two alternatives. For a small dominance, $A_{1}$ is slightly preferred to $A_{2}$, then $\alpha_{12}^{1}=(2,1/2)$ and $\alpha_{21}^{1}=(1/2,2)$. Aggregating $\alpha_{1}^{1}=(2,1/2)$ and $\alpha_{2}^{1}=(1/2,2)$, the global scores are obtained as $GS_{11}=0$ and $GS_{21}=0.22$. Thus, $PI_{1}(A_{1},A_{2})=0.22$. Therefore, we let δ<0.22. If $PI_{j}(A_{i},A_{k})\geq$ 0.22, there is significant preference relation between $A_{i}$ and $A_{k}$.

We determine the PIR relations among alternatives based on the comprehensive preference intensity $PI(A_{i},A_{k})$. We further introduce an incomparability threshold θ. The thresholds μ and θ are determined by the value of δ through analyses on the PIR relations:

(1) Based on the Pareto optimality theory, if for n−1 criteria, $\sum_{j=1}^{n-1}PI_{j}(A_{i},A_{k})-\sum_{j=1}^{n-1}PI_{j}(A_{k},A_{i})=0$, and for the nth criterion, $\max(PI_{n}(A_{i},A_{k}),$ $PI_{n}(A_{k},A_{i}))=\delta$, then $A_{i}\;P\;A_{k}$. Suppose that the same objective weight is assigned to each criterion. Then, $\left|\Delta PI(A_{i},A_{k})\right|=|PI_{n}(A_{i},A_{k})-$ $PI_{n}(A_{k},A_{i})|=\delta /n$. As this is the minimal case that $A_{i}\;P\;A_{k}$, we let μ=δ/n to distinguish the preference relation based on the net preference intensities of pairwise alternatives. We give a simple example to shown the preference relation between $A_{1}$ and $A_{2}$ in Table 1.

(2) If $\left|\Delta PI(A_{i},A_{k})\right|<\mu$ and $\max(PI_{j}(A_{i},A_{k}),PI_{j}(A_{k},A_{i}))<\delta$ for all criteria, then $A_{i}\;I\;A_{k}$. Suppose that each criterion has the same weight. Then, $\max(PI(A_{i},A_{k}),PI(A_{k},A_{i}))<\frac{n-1}{2}\times \frac{\delta }{n}+\frac{\delta }{n}=\frac{(n+1)\delta }{2n}$ if n is odd; max(PI $\left(A_{i},A_{k}),PI(A_{k},A_{i})\right)$ $<\frac{n}{2}\times \frac{\delta }{n}=\frac{\delta }{2}$ if n is even. Thus, we let θ=(n+1)δ/2n if n is odd, and θ=δ/2 if n is even. Table 2 and Table 3 show the indifference relation between $A_{1}$ and $A_{2}$ when n is odd and even respectively.

(3) For the incomparability relation $A_{i}\;P\;A_{k}$, $\left|\Delta PI(A_{i},A_{k})\right|<\mu$ and $\max(PI(A_{i},A_{k}),PI(A_{k},A_{i}))\geq \theta$. Table 4 shows the incomparability relation between $A_{1}$ and $A_{2}$.

Based on the above analyses, we present the test rule to establish the PIR relations in the IM-ORESTE method:

(20) $$\left\{ \begin{array}{l} \mathrm{If}\;\;\;\left|\Delta PI(A_{i},A_{k})\right|\geq \mu ,\;\;\mathrm{then}\left\{ \begin{array}{l} \Delta PI(A_{i},A_{k})>0,\;\;A_{i}\;P\;A_{k}\;\\ \Delta PI(A_{k},A_{i})>0,\;\;A_{k}\;P\;A_{i} \end{array}\right.\\ \mathrm{If}\;\;\left|\Delta PI(A_{i},A_{k})\right|<\mu ,\;\;\mathrm{then}\left\{ \begin{array}{l} \min\left(PI(A_{i},A_{k}),\;PI(A_{k},A_{i})\right)<\theta ,\;\;A_{i}\;I\;A_{k}\\ \max\left(PI(A_{i},A_{k}),\;PI(A_{k},A_{i})\right)\geq \theta ,\;\;A_{i}\;R\;A_{k} \end{array}\right. \end{array}\right.$$

where μ=δ/n, θ=(n+1)δ/2n if n is odd, and θ=δ/2 if n is even with δ<0.22.

The procedure of the IM-ORESTE method is given as follows.

**Algorithm** **2** (The IM-ORESTE method)

**Step 1.** Experts compare pairwise alternatives based on their performances under each criterion. The evaluations are expressed as IMNs $\alpha_{ik}^{j}$, i,k=1,2⋯m, j=1,2,⋯n. Establish the preference matrixes $D^{j}={\left(\alpha_{ik}^{j}\right)}_{m\times m}$, j=1,2,⋯n. The preferences of experts on pairwise criteria based on their importance are also expressed as IMNs $\alpha_{}^{jt}$, j,t=1,2,⋯n.

**Step 2.** Integrate the preference values $\alpha_{ik}^{j}$(k=1,2,⋯,m) into the IMN $\alpha_{i}^{j}$ under each criterion by Equation (10). Build the decision matrix $D={\left(\alpha_{i}^{j}\right)}_{m\times n}$. $\alpha_{}^{jt}$ are aggregated to $\alpha_{}^{j}$ by Equation (10).

**Step 3.** Determine $\alpha_{i}^{j+}$ by Equation (4) and calculate the distances $d(\alpha_{i}^{j},\alpha_{i}^{j+})$, j=1,2,⋯n, by Equations (2)–(3). Then compute the Pearson correlation coefficient $C_{jt}$ between two criteria by Equation (11). Determine the object weights $\omega_{j}^{\prime }$, j=1,2,⋯n, by Equation (12).

**Step 4.** Calculate the global score $GS_{ij}$ of alternative $A_{i}$ under criterion $c_{j}$ by Equation (15). $GS_{ij}$ is integrated by the performance of $A_{i}$ and the subjective and objective weights of $c_{j}$.

**Step 5.** Compute the utility values $U_{i}$, i=1,2⋯m, by Equation (16), based on which the weak rankings of alternatives are obtained.

**Step 6.** Derive the preference intensity $PI_{j}(A_{i},A_{k})$ by Equation (17), the comprehensive preference intensity $PI(A_{i},A_{k})$ by Equation (18) and the net preference intensity $\Delta PI(A_{i},A_{k})$ by Equation (19).

**Step 7.** Determine the value of δ which satisfies δ<0.22, and calculate the values of μ and θ. Then derive the PIR relations for pairwise alternatives based on the rules in Equation (20).

**Step 8.** Derive the strong rankings of alternatives considering both the weak rankings and the PIR relations between pairwise alternatives.

## 4. Case Study: Patients’ Prioritization of Admission

In this section, a case concerning the patients’ prioritization of admission is presented to illustrate the application of the IM-ORESTE method.

### 4.1. Case Description

HX is a large scale hospital in Chengdu, China. According to statistics, every month there are about 6000 patients waiting for beds for hospitalization in HX hospital. The average waiting time of each patient is about 2 to 3 months, and some patients may even have to wait half a year to be hospitalized. There are some serious patients among them who are in urgent need of hospitalization. HX hospital has about 200 thousand hospitalized patients every year. However, over 30% of them do not need to be hospitalized in HX hospital.

To solve this problem, in December 2011, HX hospital set up the Admission Service Center (ASC) to optimize the hospitalization process and promote the effective management of medical resources. Almost all beds are assigned by this center to help patients have a convenient and quick admission. It can assist the hospital to manage the beds efficiently so as to let the patients with serious illness be hospitalized quickly. However, many patients go to HX hospital no matter if they have a serious disease or not. The staff in ASC have to evaluate each patient to determine the severity of the illness and the priority. On this basis, the sequence of patients’ admission can be derived by ASC. In ASC, the evaluation indicators of patients’ prioritization are summarized as: $c_{1}$: the emergency degree of disease; $c_{2}$: the severity of disease; $c_{3}$: the type of medical insurance and its place of belonging; $c_{4}$: the scientific research value of disease; $c_{5}$: the waiting time; $c_{6}$: the priority for special disease; $c_{7}$: VIP (very important person).

Patients’ prioritization has attracted much attention in hospital management in recent years. For example, Ashour and Kremer [36] developed a dynamic grouping and prioritization algorithm to optimize the process of the emergency department of a hospital. By considering the implementation mechanism in practice, Solans-Domènech et al. [37] proposed a priority scoring system for patients who are waiting for elective surgery according to the defined criteria and their weights. Additionally, some scholars applied new approaches with a hesitant fuzzy linguistic term set to deal with the patients’ prioritization problem [38,39]. Zhang et al. [38] proposed general criteria to evaluate the prioritization of patients, and then applied the developed HFL-VIKOR method to solve the problem concerning the prioritization of patients waiting for beds in ASC. Sun et al. [39] dealt with the patients’ prioritization by using the proposed HFL-MABAC method.

In the past, the ASC selected the patients for hospitalization through artificial screening. However, there are many issues in this selection process, such as the factors of human intervention on beds and the complexity of the multifactor decision-making problem. There are no clear evaluation criteria. It is difficult for the staff in ASC to determine the priority of each patient. Motivated by this, we apply the IM-ORESTE method proposed in this paper to solve the patients’ prioritization in ASC.

Suppose that four patients are waiting for beds in ASC, and three experts are invited as a group to take part in the decision process. Note that the experts are selected from the staff in ASC and the doctors in HX hospital. The expert group analyzes all evaluation criteria and then gives their overall preference relation matrix in IMPR according to pairwise comparisons over the criteria, shown as:

(21) $$PR=\left(\begin{array}{ccccccc} \left(1,1\right) & \left(1,1\right) & \left(3,1/3\right) & \left(5/2,1/3\right) & \left(9/2,1/5\right) & \left(4,1/4\right) & \left(7,1/8\right)\\ \left(1,1\right) & \left(1,1\right) & \left(5/2,1/3\right) & \left(3,1/4\right) & \left(4,1/4\right) & \left(5,1/6\right) & \left(6,1/7\right)\\ \left(1/3,3\right) & \left(1/3,5/2\right) & \left(1,1\right) & \left(4/3,2/3\right) & \left(2,1/2\right) & \left(5/2,1/3\right) & \left(4,1/5\right)\\ \left(1/3,5/2\right) & \left(1/4,3\right) & \left(2/3,4/3\right) & \left(1,1\right) & \left(5/2,1/3\right) & \left(5/2,2/5\right) & \left(4,2/9\right)\\ \left(1/5,9/2\right) & \left(1/4,4\right) & \left(1/2,2\right) & \left(1/3,5/2\right) & \left(1,1\right) & \left(1,1\right) & \left(2,1/3\right)\\ \left(1/4,4\right) & \left(1/6,5\right) & \left(1/3,5/2\right) & \left(2/5,5/2\right) & \left(1,1\right) & \left(1,1\right) & \left(2,1/2\right)\\ \left(1/8,7\right) & \left(1/7,6\right) & \left(1/5,4\right) & \left(2/9,6\right) & \left(1/3,2\right) & \left(1/2,2\right) & \left(1,1\right) \end{array}\right)$$

The preferences of expert group on pairwise alternatives with respect to each criterion are expressed as IMPRs, shown as:

(22) $$D^{1}=\left[\begin{array}{cccc} \left(1,1\right) & \left(5,1/6\right) & \left(2,1/2\right) & \left(3,2/7\right)\\ \left(1/6,5\right) & \left(1,1\right) & \left(1/3,3\right) & \left(1/6,4\right)\\ \left(1/2,2\right) & \left(3,1/3\right) & \left(1,1\right) & \left(3/2,1/2\right)\\ \left(2/7,3\right) & \left(4,1/6\right) & \left(1/2,3/2\right) & \left(1,1\right) \end{array}\right],\;D^{2}=\left[\begin{array}{cccc} \left(1,1\right) & \left(2,1/2\right) & \left(9/2,1/5\right) & \left(4,1/4\right)\\ \left(1/2,2\right) & \left(1,1\right) & \left(3,1/3\right) & \left(5/2,1/4\right)\\ \left(1/5,9/2\right) & \left(1/3,3\right) & \left(1,1\right) & \left(2/3,1\right)\\ \left(1/4,4\right) & \left(1/4,5/2\right) & \left(1,2/3\right) & \left(1,1\right) \end{array}\right],\;D^{3}=\left[\begin{array}{cccc} \left(1,1\right) & \left(3,1/3\right) & \left(1/6,6\right) & \left(1/5,5\right)\\ \left(1/3,3\right) & \left(1,1\right) & \left(1/9,8\right) & \left(1/7,7\right)\\ \left(6,1/6\right) & \left(8,1/9\right) & \left(1,1\right) & \left(2,1/3\right)\\ \left(5,1/5\right) & \left(7,1/7\right) & \left(1/3,2\right) & \left(1,1\right) \end{array}\right],\;D^{4}=\left[\begin{array}{cccc} \left(1,1\right) & \left(1/3,3\right) & \left(4,1/6\right) & \left(3,1/4\right)\\ \left(3,1/3\right) & \left(1,1\right) & \left(7,1/7\right) & \left(6,1/7\right)\\ \left(1/6,4\right) & \left(1/7,7\right) & \left(1,1\right) & \left(2/3,3/2\right)\\ \left(1/4,3\right) & \left(1/7,6\right) & \left(3/2,2/3\right) & \left(1,1\right) \end{array}\right],\;D^{5}=\left[\begin{array}{cccc} \left(1,1\right) & \left(1/2,2\right) & \left(1/5,5\right) & \left(1/5,5\right)\\ \left(2,1/2\right) & \left(1,1\right) & \left(1/3,5/2\right) & \left(1/3,3\right)\\ \left(5,1/5\right) & \left(5/2,1/3\right) & \left(1,1\right) & \left(1,1\right)\\ \left(5,1/5\right) & \left(3,1/3\right) & \left(1,1\right) & \left(1,1\right) \end{array}\right],\;D^{6}=\left[\begin{array}{cccc} \left(1,1\right) & \left(5,1/6\right) & \left(2,1/2\right) & \left(3,1/4\right)\\ \left(1/6,5\right) & \left(1,1\right) & \left(1/4,3\right) & \left(1/3,2\right)\\ \left(1/2,2\right) & \left(3,1/4\right) & \left(1,1\right) & \left(2,1/4\right)\\ \left(1/4,3\right) & \left(2,1/3\right) & \left(1/4,2\right) & \left(1,1\right) \end{array}\right],\;D^{7}=\left[\begin{array}{cccc} \left(1,1\right) & \left(1/5,5\right) & \left(2,1/2\right) & \left(2,1/4\right)\\ \left(5,1/5\right) & \left(1,1\right) & \left(7,1/7\right) & \left(6,1/7\right)\\ \left(1/2,2\right) & \left(1/7,7\right) & \left(1,1\right) & \left(1/2,2\right)\\ \left(1/4,2\right) & \left(1/7,6\right) & \left(2,1/2\right) & \left(1,1\right) \end{array}\right].$$

### 4.2. Solving the Case by the IM-ORESTE Method

**Step 1.** The relevant evaluation information is given in Section 4.1.

**Step 2.** Aggregate the preference values $\alpha_{ik}^{j}$ (k=1,2,⋯,m) into the IMN $\alpha_{i}^{j}$ by Equation (10). Then, a decision matrix $D={\left(\alpha_{i}^{j}\right)}_{m\times n}$ can be derived:

$$D=\left(\begin{array}{ccccccc} \left(3.3431,0.2553\right) & \left(3.4961,0.2719\right) & \left(0.6991,1.4304\right) & \left(2.1496,0.3103\right) & \left(0.2793,3.5808\right) & \left(3.3431,0.2450\right) & \left(1.1589,0.5892\right)\\ \left(0.2131,3.8907\right) & \left(1.8238,0.4375\right) & \left(0.1795,5.3789\right) & \left(5.5582,0.1676\right) & \left(0.7116,1.3399\right) & \left(0.2431,3.0426\right) & \left(6.0859,0.1551\right)\\ \left(1.5098,0.5986\right) & \left(0.3682,2.2155\right) & \left(5.9390,0.1111\right) & \left(0.2707,3.2661\right) & \left(2.7581,0.3418\right) & \left(1.6706,0.3835\right) & \left(0.3459,2.8914\right)\\ \left(1.2093,0.5056\right) & \left(0.4286,1.6767\right) & \left(4.0982,0.2359\right) & \left(0.4548,1.9530\right) & \left(2.9257,0.3418\right) & \left(0.6152,0.9902\right) & \left(0.5386,1.4787\right) \end{array}\right)$$

**Step 3.** Calculate the Pearson correlation coefficient $C_{jt}$ by Equation (11) and then derive the objective weights of criteria according to Equation (12). Then, we obtain $\omega_{1}^{\prime }$ = 0.1261, $\omega_{2}^{\prime }$ = 0.1321, $\omega_{3}^{\prime }$ = 0.1418, $\omega_{4}^{\prime }$ = 0.1474, $\omega_{5}^{\prime }$ = 0.1727, $\omega_{6}^{\prime }$ = 0.1239, $\omega_{7}^{\prime }$ = 0.1560. Additionally, the score values $s(\alpha_{i}^{j})$ of $\alpha_{i}^{j}$ can be calculated by Equation (2) and shown in Table 5.

**Step 4.** Calculate the global score $GS_{ij}$ by Equation (15) and the results are given in Table 6.

**Step 5.** The utility values of patients are calculated as $U_{1}$ = 0.3013, $U_{2}$ = 0.3860, $U_{3}$ = 0.3581 and $U_{4}$ = 0.3541. Thus, we obtain $A_{1}>A_{4}>A_{3}>A_{2}$.

**Step 6.** Calculate $PI_{j}(A_{i},A_{k})$, $PI(A_{i},A_{k})$ and $\Delta PI(A_{i},A_{k})$ from Equations (17)–(19). The results are shown in Table 7 and Table 8.

**Step 7.** Predefine that δ=0.1845, based on which, the values of μ and θ can be determined as μ=0.0264 and θ=0.1054. By Equation (20), the PIR relations of the alternatives are: $A_{1}PA_{2}$, $A_{1}PA_{3}$, $A_{1}PA_{4}$, $A_{3}PA_{2}$, $A_{4}PA_{2}$, $A_{3}IA_{4}$.

**Step 8.** Based on the weak ranking and the PIR relations of the alternatives, we can obtain the final ranking of alternatives as $A_{1}>A_{3}∼A_{4}>A_{2}$.

### 4.3. Comparative Analyses and Discussions

In this section, a detailed comparative analysis between the IM-ORESTE method and the existing MCDM methods is presented to illustrate the effectiveness and superiority of our proposed method.

Considering that the crisp weights of criteria are the basis of most MCDM methods, we translate the subjective weights evaluated by DMs to crisp weights by

(23) $${\tilde{w}}_{j}^{\prime \prime }=\frac{s\left(\alpha^{j}\right)}{\sum_{j=1}^{n}s\left(\alpha^{j}\right)}$$

Then we apply Equation (22) to integrate the crisp subjective weight and the objective weight calculated from Equation (12) of each criterion into a collective one.

(24) $$w_{j}=\frac{\sqrt{w_{j}^{\prime }{\tilde{w}}_{j}^{\prime \prime }}}{\sum_{j=1}^{n}\sqrt{w_{j}^{\prime }{\tilde{w}}_{j}^{\prime \prime }}}$$

By Equation (22), we obtain $w_{1}$ = 0.3108, $w_{2}$ = 0.3092, $w_{3}$ = 0.1338, $w_{4}$ = 0.1258, $w_{5}$ = 0.0567, $w_{6}$ = 0.0426, $w_{7}$ = 0.0211. We respectively apply the IM-TOPSIS method, IM-VIKOR method and IM-PROMETHEE method to solve this problem, and the calculation results are shown in Table 9.

From Table 9, we know that the patient $A_{1}$ has the highest priority compared with other patients. There are some differences in the ranking of patients when we apply different MCDM methods. For the IM-TOPSIS method, the ranking of patients is $A_{1}>A_{3}>A_{4}>A_{2}$. For the IM-VIKOR method, if we take λ = 0.1, 0.7, 0.8 and 0.9, the ranking of patients is $A_{1}>A_{3}>A_{4}>A_{2}$; while the ranking of patients is $A_{1}>A_{4}>A_{3}>A_{2}$ when λ = 0.2, 0.3, 0.4, 0.5, 0.6 (see Table 10). In addition, we obtain $A_{1}>A_{3}>A_{4}\;\;R\;\;A_{2}$ by applying the IM-PROMETHEE method, but we cannot determine the relation between the patients $A_{2}$ and $A_{4}$.

As we know, both the IM-TOPSIS method and the IM-VIKOR method are based on the overall utility values of alternatives. Neither the IM-TOPSIS method nor the IM-VIKOR method can derive the PIR relations among the alternatives. The IM-TOPSIS method fails to accurately describe the indifference relation between the patients $A_{3}$ and $A_{4}$, although there is little difference between the relative coefficient values of them. From Table 10, we also find that there is little difference between the overall utility values of the patients $A_{3}$ and $A_{4}$. But the IM-VIKOR method also fails to accurately distinguish the relation between them.

Some theoretical defects exist in the IM-PROMETHEE method. There is indifference relation between $A_{i}$ and $A_{j}$, if and only if both the positive outranking flow and the negative outranking flow of them are equal. The IM-PROMETHEE method is based on the outranking flows of alternatives but not the preference relations of alternatives over the criteria. Therefore, the calculation result is questionable to some extent.

For the IM-ORESTE method, it can overcome the defects discussed above. In other words, it is more reasonable than the IM-TOPSIS method, the IM-VIKOR method, and the IM-PROMETHEE method in practice. Specifically, the IM-ORESTE method has the following advantages:

(1) Both the objective and subjective weights of criteria are considered in the IM-ORESTE method. In addition, the correlation coefficient-based weight determining method can avoid the effects of correlated criteria on the final result.

(2) The PIR relations are derived from the IMPR of alternatives but not from the outranking flows. The preference, indifference, and incomparability relations among the alternatives are determined rationally based on the scientific calculation process.

## 5. Conclusions

Compared with the primary medical institutions, the public hospitals in China face a huge sickbed crisis. An irrational order of hospitalization often leads to worsening of the disease. In recent years, there has been a dramatic increase of patient dissatisfaction on the admission process in China. A reasonable method is needed to rank a patient for hospitalization based on multiple conflict criteria. This is a typical MCDM problem. This paper proposed the IM-ORESTE method to rank the patients according to seven predefined evaluation criteria. The advantages of this method compared with other existing MCDM methods are summarized as follows:

1. Experts are allowed to give their opinions by pairwise comparisons of alternatives, which are easy to determine. The uncertain evaluations of experts can be fully reflected by the IMSs in terms of the membership degree, non-membership degree, and uncertainty degree.
2. Based on the correlation coefficient-based objective weight determining method, the biased results caused by the highly-related criteria can be avoided in solving MCDM problems.
3. Experts’ preferences on the importance of criteria are also considered. The fuzzy subjective weights of the criteria are not required to translate into crisp weights, which can avoid information loss.
4. A reliable result is ensured by the conflict analysis to distinguish the PIR relations between alternatives.

On this basis, we applied the proposed IM-ORESTE method to solve the problem concerning patients’ prioritization of hospitalization in China. The results showed that it can be used to deal with the complex MCDM problem in which the weights of criteria were unknown and both the qualitative and quantitative criteria existed simultaneously.

However, there are some limitations in the proposed MCDM method. When applying the proposed method to handle this kind of problem concerning hospitalization, it may cost manual effort since heavy work exists in the process of pairwise comparison between patients. Thus, in the future, we will continue to improve this method via some computer aid techniques to make it timesaving and laborsaving. In addition, we will focus on setting up a systematic criterion to rank the patients for hospitalization. It would be valuable to apply the developed IM-ORESTE method to other medical management problems. Extending the improved ORESTE method to other types of evaluation models [34,40] should be interesting as well.

## Figures and Tables

**Table 1:** The minimal situation for the preference relation between $A_{1}$ and $A_{2}$.

Preference	$c_{1}$	$c_{2}$	$c_{3}$	$c_{4}$
$A_{1}$	(2,1/2)	(1/2,2)	(1,1)	(1/3,3)
$A_{2}$	(1/2,2)	(2,1/2)	(1,1)	(3,1/3)

**Table 2:** The indifference relation between $A_{1}$ and $A_{2}$ if n is odd.

Preference	$c_{1}$	$c_{2}$	$c_{3}$	$c_{4}$	$c_{5}$
$A_{1}$	(2/3,3/2)	(3/2,2/3)	(3/4,4/3)	(4/3,3/4)	(3/2,3/5)
$A_{2}$	(3/2,2/3)	(2/3,3/2)	(4/3,3/4)	(3/4,4/3)	(3/5,3/2)

**Table 3:** The indifference relation between $A_{1}$ and $A_{2}$ if n is even.

Preference	$c_{1}$	$c_{2}$	$c_{3}$	$c_{4}$
$A_{1}$	(2/3,3/2)	(3/2,2/3)	(3/4,4/3)	(4/3,3/4)
$A_{2}$	(3/2,2/3)	(2/3,3/2)	(4/3,3/4)	(3/4,4/3)

**Table 4:** The incomparability relation between $A_{1}$ and $A_{2}$.

Preference	$c_{1}$	$c_{2}$	$c_{3}$	$c_{4}$
$A_{1}$	(5,1/5)	(1/5,5)	(1/6,6)	(6,1/6)
$A_{2}$	(1/5,5)	(5,1/5)	(6,1/6)	(1/6,6)

**Table 5:** The score values $s(\alpha_{i}^{j})$.

$s(\alpha_{i}^{j})$	$C_{1}$	$C_{2}$	$C_{3}$	$C_{4}$	$C_{5}$	$C_{6}$	$C_{7}$
$A_{1}$	13.0933	12.8569	0.4888	6.9264	0.0780	13.6443	1.9669
$A_{2}$	0.0548	4.1684	0.0334	33.1676	0.5311	0.0799	39.2361
$A_{3}$	2.5223	0.1662	53.4507	0.0829	8.0693	4.3565	0.1196
$A_{4}$	2.3921	0.2556	17.3703	0.2329	8.5598	0.6213	0.3642

**Table 6:** The global scores of each alternative.

$GS_{ij}$	$C_{1}$	$C_{2}$	$C_{3}$	$C_{4}$	$C_{5}$	$C_{6}$	$C_{7}$
$A_{1}$	0.0000	0.0010	0.0619	0.0325	0.0835	0.0404	0.0820
$A_{2}$	0.0558	0.0139	0.0910	0.0234	0.0653	0.0660	0.0706
$A_{3}$	0.0173	0.0478	0.0209	0.0754	0.0522	0.0427	0.1019
$A_{4}$	0.0206	0.0446	0.0270	0.0638	0.0521	0.0526	0.0933

**Table 7:** The comprehensive preference intensities.

$PI(A_{i},A_{k})$	$A_{1}$	$A_{2}$	$A_{3}$	$A_{4}$
$A_{1}$	0.0000	0.1234	0.1293	0.1191
$A_{2}$	0.0387	0.0000	0.1173	0.0938
$A_{3}$	0.0725	0.1451	0.0000	0.0194
$A_{4}$	0.0663	0.1257	0.0235	0.0000

**Table 8:** The net preference intensities.

$\Delta PI(A_{i},A_{k})$	$A_{1}$	$A_{2}$	$A_{3}$	$A_{4}$
$A_{1}$	0.0000	0.0847	0.0568	0.0528
$A_{2}$	−0.0847	0.0000	−0.0279	−0.0319
$A_{3}$	−0.0568	0.1730	0.0000	−0.0040
$A_{4}$	−0.0528	0.0319	0.0040	0.0000

**Table 9:** Comparison between our proposed method and the existing methods.

Methods	Coefficient	$A_{1}$	$A_{2}$	$A_{3}$	$A_{4}$	The Ranking Order
IM-TOPSIS	The relative coefficient	0.7755	0.3611	0.4845	0.4820	$A_{1}>A_{3}>A_{4}>A_{2}$
IM-VIKOR	$IMGU_{i}$	0.1433	0.4023	0.3267	0.3422	
	$IMIR_{i}$	0.0778	0.1947	0.1584	0.1477	
	$IMC_{i}$ (λ=0.5)	0	1	0.6988	0.6830	$A_{1}>A_{4}>A_{3}>A_{2}$
IM-PROMETHEE	$S_{P}\left(A_{i}\right)$ Positive outranking flow	18.6450	4.7028	14.1578	2.6417	
	$S_{N}\left(A_{i}\right)$ Negative outranking flow	0.8407	25.1155	8.8524	9.1867	
	$S\left(A_{i}\right)$ Net outranking flow	17.8043	−20.4127	5.3054	−6.5451	$A_{1}>A_{3}>A_{4}\;\;R\;\;A_{2}$
In this paper	Utility values of the patients $U_{i}$	0.3013	0.3860	0.3581	0.3541	$A_{1}>A_{3}∼A_{4}>A_{2}$

**Table 10:** The calculation results of the IM-VIKOR method.

		$A_{1}$	$A_{2}$	$A_{3}$	$A_{4}$	The Ranking Order
$IMGU_{i}$		0.1433	0.4023	0.3267	0.3422	$A_{1}>A_{3}>A_{4}>A_{2}$
$IMIR_{i}$		0.0778	0.1947	0.1584	0.1477	$A_{1}>A_{4}>A_{3}>A_{2}$
$IMC_{i}$	λ=0.1	0	1	0.6913	0.6149	$A_{1}>A_{4}>A_{3}>A_{2}$
	λ=0.2	0	1	0.6932	0.6319	$A_{1}>A_{4}>A_{3}>A_{2}$
	λ=0.3	0	1	0.6951	0.6489	$A_{1}>A_{4}>A_{3}>A_{2}$
	λ=0.4	0	1	0.6969	0.6659	$A_{1}>A_{4}>A_{3}>A_{2}$
	λ=0.5	0	1	0.6988	0.6830	$A_{1}>A_{4}>A_{3}>A_{2}$
	λ=0.6	0	1	0.7007	0.7000	$A_{1}>A_{4}>A_{3}>A_{2}$
	λ=0.7	0	1	0.7025	0.7170	$A_{1}>A_{3}>A_{4}>A_{2}$
	λ=0.8	0	1	0.7044	0.7340	$A_{1}>A_{3}>A_{4}>A_{2}$
	λ=0.9	0	1	0.7062	0.7510	$A_{1}>A_{3}>A_{4}>A_{2}$
